# Metabolomic Insights into Attention Deficit Hyperactivity Disorder: A Scoping Review

**DOI:** 10.3390/metabo15020133

**Published:** 2025-02-16

**Authors:** Maria Jose Muñoz-Zabaleta, Nicolás Garzón Rodríguez, Luis Eduardo Díaz-Barrera, Maria Fernanda Quiroz-Padilla

**Affiliations:** 1 Laboratorio de Bases Biológicas del Comportamiento, Facultad de Psicología y Ciencias del Comportamiento, Universidad de La Sabana, Chía 250001, Colombia; mariamuza@unisabana.edu.co (M.J.M.-Z.); nicolas.garzon@unisabana.edu.co (N.G.R.); 2 Doctorado en Psicología, Facultad de Psicología y Ciencias del Comportamiento, Universidad de La Sabana, Chía 250001, Colombia; 3 Doctorado en Biociencias, Facultad de Ingeniería, Universidad de La Sabana, Chía 250001, Colombia; 4 Bioprospecting Research Group, Facultad de Ingeniería, Universidad de La Sabana, Chía 250001, Colombia; luis.diaz1@unisabana.edu.co

**Keywords:** metabolomics, metabolites, lipidomic, biomarkers, ADHD

## Abstract

**Background /Objectives** Attention deficit hyperactivity disorder (ADHD) is the most common neurodevelopmental condition, and symptoms persist into adulthood. Its etiology, though recognized as multifactorial, is still under discussion. Metabolomics helps us to identify pathways associated with functional and structural changes that may be related to symptomatology. This study aimed to characterize potentially altered metabolic pathways and associated biochemical reactions in ADHD. **Methods:** A scoping review of experimental research was conducted using PubMed, Web of Science, and Scopus using PRISMA ScR. Fifty-five studies were eligible for data extraction, of which fifteen met the criteria for inclusion in the review. Subsequently, the identified metabolites were analyzed in the context of the literature to recognize possible discordant pathways in the disorder. **Results:** Two groups of relevant neuromodulators of ADHD were found: precursors of monoamines and polyunsaturated fatty acids. The literature was reviewed to discover potential implicated pathways and new metabolites of interest. **Conclusions:** The study of ADHD biomarkers should focus on measuring precursor, intermediate, and final metabolites of polyunsaturated fatty acids and monoamines in panels or through untargeted analysis to improve the understanding of the pathology and individualization of treatments.

## 1. Introduction

Attention deficit hyperactivity disorder (ADHD) is a neurodevelopmental condition characterized by symptoms of attention difficulties, hyperactivity, and impulsivity, this is the most common neurodevelopmental disorder, with a prevalence in children of approximately 5% [1], and its symptoms persist in approximately 2.5% of adults [2,3]. Its etiology is considered multifactorial due to the interaction of genetic variants [4] and environmental [5], infectious and metabolic factors [6,7] that produce functional and structural alterations in the brain. These changes are reflected in the altered metabolism of amino acids [8,9], fatty acids [10], and minerals [10], which affect brain function [11,12]. However, the subjective nature of symptom reporting and the absence of definitive biomarkers complicate accurate diagnosis, highlighting the challenges in identifying the underlying neurobiological causes of ADHD and distinguishing it from other conditions [13].

Metabolomics allows us to obtain information about the underlying biochemical processes in various neuropsychiatric conditions, helping us to explain the pathophysiology of diseases, recognize biomarkers [14], therapeutic targets, and individualize the treatments. In patients with ADHD, differences in metabolic profiles compared to controls have been identified, which could aid in diagnosing and monitoring the disease and in differentiating the clinical presentations [15]. Low levels of amino acids such as tyrosine and phenylalanine, precursors of dopamine (DA) [16], and tryptophan, a precursor of serotonin [17,18,19], have been found in individuals with ADHD.

Moreover, there is an increase in waste metabolites of tyrosine, such as p-hydroxyphenylpyruvate and p-hydroxyphenyl acetate, which produce neuronal toxicity and are associated with an increase in free radicals, activation of the immune system, and damage to the blood–brain barrier [20]. This inflammation, due to the immune system’s response mediated by proinflammatory cytokines, can induce structural and/or functional changes in the brain [6,7].

The supplementation of fatty acids in diagnosed children has shown changes in inflammatory markers and behavioral and cognitive profiles [21]. These fatty acids play crucial roles in the central nervous system (CNS), acting as an energy source for neurons, facilitating the synthesis of phospholipids, prostaglandins, and leukotrienes necessary for brain function [22], participating in the regulation of neurotransmitters [23], and exhibiting anti-inflammatory properties in the body [24]. An imbalance in omega-6/omega-3 fatty acid levels can alter cell membrane properties and increase the production of inflammatory mediators due to the increase in eicosanoids, such as prostaglandins, leukotrienes, and thromboxanes [21]. Additionally, deficient levels of eicosapentaenoic acid (EPA) and docosahexaenoic acid (DHA) have been recurrently observed in individuals with ADHD [21,24,25].

The recent literature in the field of ADHD metabolomics is advancing toward a more comprehensive approach, focusing on the identification of alterations in specific metabolic pathways rather than solely on individual metabolites [26]. This shift in perspective is essential, as it enables an enhanced understanding of the underlying biological mechanisms of ADHD, highlighting metabolic pathways related to neurotransmission, oxidative stress, and amino acid metabolism, as observed in recent studies involving the tryptophan–serotonin axis and the arginine/nitric oxide pathway [27,28]. Unlike other conditions, such as cancer [29], where exhaustive literature reviews have been conducted on altered metabolic pathways specific to the condition, in the case of ADHD, a detailed theoretical review of the potential metabolic pathways associated with neuromodulators reported individually in previous studies has yet to be carried out.

This scoping review aimed to characterize potentially altered metabolic pathways and associated biochemical reactions in ADHD. Understanding metabolomics in the disorder could provide valuable insights into its underlying biological mechanisms, potentially contributing to improved diagnostic precision and the development of targeted intervention strategies.

## 2. Materials and Methods

### 2.1. Data Search, Eligibility Criteria, and Study Selection

A literature review of studies published from January 2004 to November 2024 was carried out. The following analytical techniques employed in metabolomics were considered eligible: gas chromatography (GC), liquid chromatography (LC) coupled with mass spectrometry (MS), and/or nuclear magnetic resonance (NMR) in different biofluids (blood, saliva, and urine). Only studies published in English were reviewed. All reviews, studies without a control group, and those measuring non-dietary metabolites were excluded. The literature search was performed in the PubMed, Web of Science, and Scopus databases, combining multiple terms related to “metabolomics” and “attention deficit hyperactivity disorder”. The search strings are presented in Supplementary Table S1. Then, two independent researchers reviewed the titles and abstracts of the identified references, resolving inconsistencies in a consensus review.

### 2.2. Data Extraction and Quality Assessment

Information was collected from each included study on the following aspects: authors, year of publication, country of publication, study design, sample size and age, type of ADHD, neuropsychological tests used, biological sample (biofluid), metabolomic techniques, and main findings.

Subsequently, with the metabolites found, a pathway analysis was conducted using the Metabolic Atlas database. Finally, an exploratory search was performed to find literature reporting alterations in enzymatic or genetic pathways.

## 3. Results

**Figure 1:**
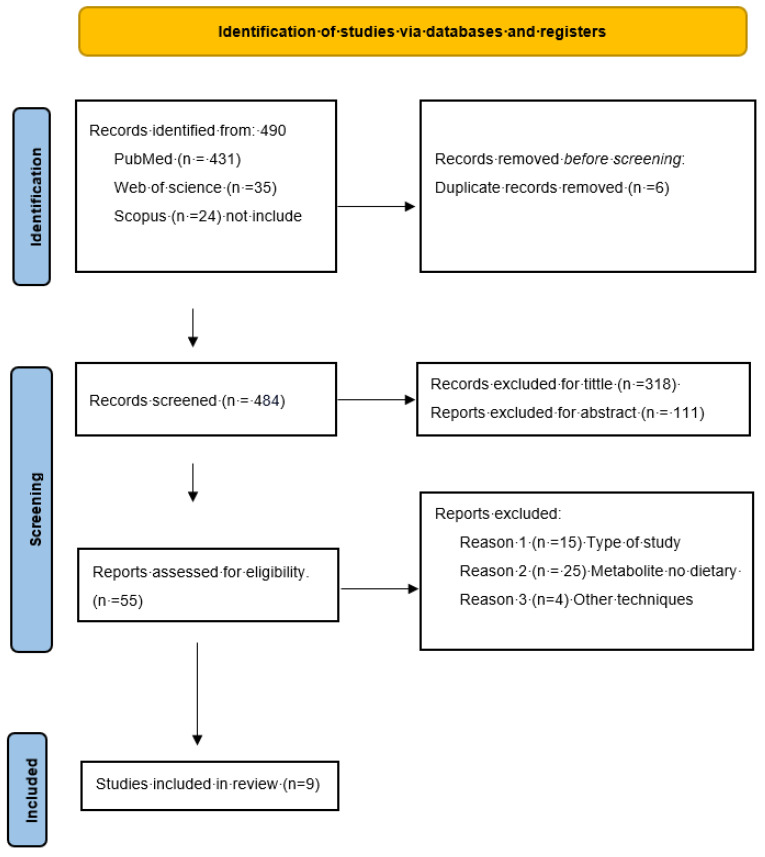
PRISMA flow diagram. Flowchart of the systematic literature search according to PRISMA guidelines. Modified from [39].

A total of 490 references were identified according to the search criteria in PubMed (n = 431), Web of Science (n = 35), and Scopus (n = 24) up to November 2024. An initial title screening was performed, excluding 318 studies that did not meet the inclusion criteria and removing 6 duplicates. The abstracts of 166 studies were analyzed, and 55 were preselected. After a full-text review was conducted, 10 of these were chosen for data extraction. Of the remaining 44 studies, 13 did not include a control group, 4 did not use high-resolution techniques, 2 were literature reviews, and 26 studied metabolites not relevant to our objectives, such as thiols, nitric acid, and lipid transporters. Of the 10 selected studies, an article using a urine sampling technique was excluded to facilitate comparison and ensure methodological consistency among the included studies, thereby ensuring greater methodological uniformity, reducing data heterogeneity, and supporting more robust and standardized conclusions on the analyzed metabolites. This information is further detailed in Figure 1. A total of nine studies evaluating metabolites in blood remained for further analysis. Of these, eight focused on pediatric populations (under 18 years), and only two included metabolic data from adults. Two studies were randomized clinical trials with preintervention metabolite reports; the rest were observational case–control studies. All the articles used self-reported or parent-reported scales to confirm the diagnosis.

Four studies assessed amino acid levels in blood samples from children with ADHD. Three of these employed liquid chromatography–tandem mass spectrometry (LC–MS/MS), and one used Infrared spectroscopy with partial least squares discriminant analysis (PLS-DA). All samples corresponded to pediatric populations. Although the results were heterogeneous, they consistently reported low levels of neurotransmitter precursors such as dopamine and serotonin (see Table 1).

**Table 1:** Characteristics of the studies included in this scoping review.

Study	Type of Study	Sample	Age	Metabolites	Technique	Results	
						General	Main
Bergweff; 2016 Netherlands [30]	Exploratory observational case–control	83 ADHD/72 Controls	6–13 years	Amino acids	LC-MS/MS	(−) Tryptophan (−) Tyrosine (−) Phenylalanine	No differences in amino acids between the control group and ADHD group, but an increased concentration of phenylalanine in blood raises the risk of an ADHD diagnosis.
Ildiz, 2021 Turkey [31]	Exploratory observational case–control	30 ADHD/29 Controls	6–14 years	Amino acids	Infrared spectroscopy	(−) Tryptophan (↓) Tyrosine (−) Phenylalanine	Increased overall protein profile in ADHD, with a decrease in tyrosine and phenylalanine levels.
Wang 2021 Taiwan [32]	Exploratory observational case–control	58 ADHD/38 Controls	<18 years	Amino acids	LC-MS/MS	(↓) Tryptophan (−) Tyrosine (↓) Phenylalanine (↑) Hippuric (↑) Acid hydroxylysine (↑) Hypoxanthine (↑) Phenylleucine (↑) Phosphoethanolamine	Profile showing an increase in Guanosine, O-Phosphoethanolamine, Phenylleucine, Hypoxanthine, 4-Aminohippuric acid, 5-Hydroxylysine, and L-Cystine, along with a decrease in Gentisic acid and Tryptophyl-phenylalanine. This panel may have good accuracy in ADHD diagnosis, with an area under the curve (AUC) of 0.923.
Chen, 2004 Russia [33]	Exploratory observational case–control	68 ADHD/38 Controls	4–12 years	Minerals Vitamins Amino acids	Gas chromatography	(↑) Linoleic acid (↓) Arachidonic acid (↓) Docosahexaenoic acid (↓) Alpha−linolenic acid (↑) Oleic acid (↓) Nervonic acid	The levels of linolenic acid and oleic acid were higher in the ADHD group compared to the control group. The concentrations of nervonic acid, linoleic acid, arachidonic acid, and docosahexaenoic acid were significantly lower in the ADHD group.
Young, 2004 Canada [34]	Exploratory observational case–control	37 ADHD/35 Controls	18–65 years	Fatty acids	Gas chromatography	(↓) Linoleic acid (↓) Arachidonic acid (↓) Docosahexaenoic acid (↓) Alpha−linolenic acid (↓) Eicosapentaenoic acid (↓) Oleic acid (↓) Nervonic acid	Lower levels of saturated fatty acids, monounsaturated, polyunsaturated, total omega-6, DHA, and DPA were observed in both erythrocyte membranes and serum phospholipids. No association was found between ADHD symptoms and fatty acid levels.
Joshi 2006 India [35]	Randomized clinical trial	30 ADHD/30 Controls	6–9 years	Fatty acids	Gas chromatography	(−) Linoleic acid (−) Arachidonic acid (−) Docosahexaenoic acid (−) Alpha−linolenic acid (−) Oleic acid (−) Nervonic acid	No differences in baseline measurements of fatty acids and antioxidants were observed between cases and controls.
Laasonen 2009 Finland [36]	Exploratory observational case–control	ADHD/Controls 26 ADHD/36 Controls	10–55 years	Fatty acids	Gas chromatography	(↑) Linoleic acid (−) Docosahexaenoic acid (−) Alpha−linolenic acid (−) Oleic acid	Association between PUFA n-6 levels and executive function, with significant associations between n-6/n-3 levels and mental flexibility, as well as between n-6 PUFA and inhibition, MFA, PUFA, n-6 PUFA, and planning.
Gustafsson 2010 Sweden [37]	Randomized clinical trial	37 ADHD/36 Controls	7–12 years	Fatty acids	Gas chromatography	(↓) Docosahexaenoic acid (↓) Alpha−linolenic acid (−) Eicosapentaenoic acid	No differences between cases and controls except for docosahexaenoic acid.
Yonezawa 2018 Austria [38]	Exploratory observational case–control	ADHD/Controls 24 ADHD/24 Controls	Not reported	Fatty acids	Gas chromatography	(↓) Docosahexaenoic acid (↓) Alpha−linolenic acid	Lower EPA and DHA levels compared to adult controls, and no correlation with ADHD symptoms.

(↑) The metabolite was found to be increased in the ADHD group compared to controls. (−) There were no differences between the groups. (↓) The metabolite was found to be decreased in the ADHD group.

Seven papers measured fatty acids in patients with ADHD through blood samples, while one study used urine samples, which was excluded from the analysis to reduce heterogeneity. However, six studies identified alterations in fatty acid profiles, showing consistent evidence of a decrease in polyunsaturated fatty acids (mainly omega-3) in patients with ADHD, as well as nervonic (omega-9) and arachidonic acids (omega-6). However, the results were not equally homogeneous for linoleic acid (Omega-6) and oleic acid (Omega-9).

This review aimed to characterize potentially altered metabolic pathways and associated biochemical reactions in ADHD. Two groups of neuromodulators relevant to ADHD were identified: monoamine precursor amino acids and polyunsaturated fatty acids. This finding is consistent with what has been reported in a recent review on altered metabolic processes in ADHD, which described a lipid imbalance, amino acid dysregulation, neurotransmitter dysfunction, and kynurenine pathway dysregulation [38]. The potential implications for the pathophysiology of ADHD are described below.

### 3.1. Amino Acids and ADHD

Due to monoaminergic hypotheses in mental disorders, the levels of the aromatic amino acids tryptophan and tyrosine have been of particular interest in ADHD research, with the hypothesis that their deficiencies could reduce the synthesis of serotonin and dopamine. Studies with small sample sizes have reported lower levels of phenylalanine, tyrosine, and tryptophan [40]. However, the evidence is inconsistent, and a possible explanation for this discrepancy could be alterations in the pathways of these amino acids rather than their levels themselves. Next, we will explain the alterations found in each of these pathways.

#### 3.1.1. Tryptophan Pathway and ADHD

Tryptophan is an essential amino acid and a precursor of serotonin, a neurotransmitter implicated in mood. It is converted into 5-hydroxytryptophan (5-HTP) by the enzyme tryptophan hydroxylase 1 (TPH1) in peripheral tissues and tryptophan hydroxylase 2 (TPH2) in the central nervous system, both of which are rate-limiting for serotonin synthesis [40,41]. Polymorphisms in the tryptophan hydroxylase 2 (TPH2) loci, identified as a susceptibility gene, have been reported in individuals with ADHD [35]. The G allele has been associated with dysfunction in the prefrontal cortex [42,43] and altered reactivity [44], while the T allele has lower reward insatiability [45].

5-HTP is subsequently converted to serotonin by the enzyme aromatic L-amino acid decarboxylase (DDC) [46]. The DDC gene is associated with motor symptoms such as hypotonia and/or autonomic dysfunctions such as excessive sweating, thermal instability, and sleep disturbances [47], all of which are highly comorbid in people with ADHD [48].

In the pineal gland, which is regulated by the circadian cycle, serotonin is converted to N-acetylserotonin (NAS) by the enzyme arylalkylamine N-acetyltransferase (AANAT) and subsequently to melatonin by the enzyme N-acetylserotonin O-methyltransferase (ASMT). Melatonin is important for regulating sleep quality, protecting cells from oxidative damage, and reducing inflammation [49]. An altered mechanism of this hormone has been reported in ADHD patients, identifying a splice site mutation in ASMT and a nonsense mutation in *MNTR1A* (a gene encoding the melatonin receptor 1A, abundant in the prefrontal cortex and striatum) that nullifies the activity of the ASMT and AANAT enzymes and could explain the high comorbidity with sleep disorders reported in more than 25% of people with ADHD [50]. A recent systematic review identified this indolamine pathway as a potential biomarker of ADHD [38]. Furthermore, in animal model studies with 39, XY*O mice, which have an Xp22.3 deletion and a relevant neurodevelopmental disorder phenotype, ASMT enzyme deficiency was also found to be associated with hyperactive symptoms, inflammatory responses, and altered synapses [51].

Another tryptophan metabolism is the kynurenine pathway, which metabolizes 95% of tryptophan. In this route, the enzymes tryptophan 2,3-dioxygenase (TDO) in the liver and indoleamine 2,3-dioxygenase (IDO) in the brain catalyze the conversion of the compound to kynurenine (KYN). KYN is subsequently metabolized through two pathways: a neuroprotective way modulated by kynurenine aminotransferase (KAT), which converts it into kynurenic acid (KYNA), an antagonist of N-methyl-D-aspartate (NMDA) receptors in the brain that protects neurons from excitotoxic damage by overexcitation and thus plays a role in immune system regulation [52], and a neurotoxic route modulated by kynurenine 3-monooxygenase (KMO), which degrades the metabolite into 3-hydroxykynurenine (3HK). 3HK is then converted by kynureninase (KYNU) into 3-hydroxyanthranilic acid (3-HAA) and finally by 3-hydroxyanthranilate 3,4-dioxygenase (HAAO) into quinolinic acid (QUIN), a toxic metabolite (an NMDA receptor antagonist), adenosine triphosphate (ATP), adenine dinucleotide (NAD), and picolinic acid (PIC), the latter being neuroprotective [53].

When exposed to physical or psychological stress, proinflammatory cytokines activate the hypothalamic–pituitary–adrenal (HPA) axis, promoting the secretion of glucocorticoids, which increases TDO activity [54]. This inflammatory mediation enhances KMO activity, creating an imbalance that results in increased QUIN production [55]. This imbalance has also been reported to be due to KAT dysfunction [56].

In line with current evidence, we propose that a proinflammatory state could explain the imbalance in tryptophan levels and its metabolites in ADHD. This may arise due to an increase in the availability of tryptophan, which is converted into proinflammatory kynurenines instead of serotonin or melatonin, due to enzymatic difficulties in its conversion at the brain level. These imbalances may be related to TPH2, DDC, and ASMT enzyme dysfunctions. Additionally, physical and psychological stress associated with the condition can exacerbate this process, promoting additional inflammatory states through HPA axis dysfunction.

**Figure 2:**
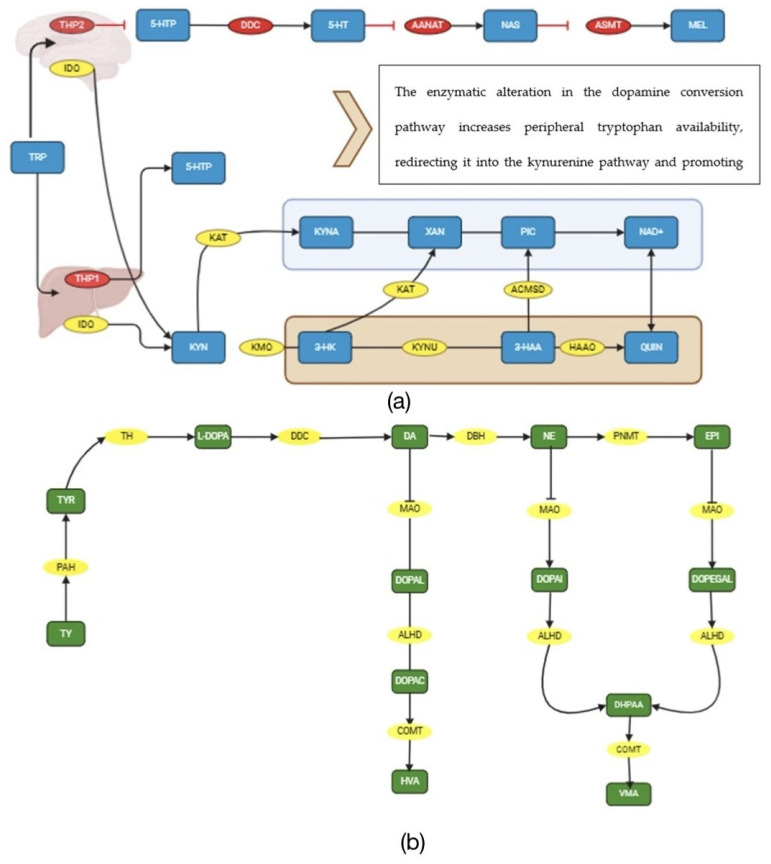
Schematic of the metabolic pathway of tryptophan and phenylalanine in ADHD. (**a**) Tryptophan metabolism pathway. Metabolites (blue rectangle): 5-HTP—5-Hydroxytryptophan, 5-HT—5-Hydroxytryptamine (serotonin), NAS—N-acetylserotonin, MEL—melatonin, KYN—kynurenine, 3-HK—3-hydroxykynurenine, 3-HAA—3-hydroxyanthranilic acid, QUIN—quinolinic acid, KYNA—kynurenic acid, XAN—Xanthurenic Acid, PIC—picolinic acid, NAD+—Nicotinamide Adenine Dinucleotide. Enzymes (yellow and red ovals): IDO—indoleamine 2,3-dioxygenas, THP1—tryptophan hydroxylase 1, THP2—tryptophan hydroxylase 2, KAT—kynurenine aminotransferase, KMO—kynurenine 3-monooxygenas, KYNU—kynureninase, ACMSD—Aminocarboxymuconate Semialdehyde Decarboxylase, HAAO—3-hydroxyanthranilate 3,4-dioxygenase. (**b**) Tyrosine metabolism pathway. Stars indicate damage reported in the literature to enzymes in this pathway. Metabolites (green rectangle): Phe—phenylalanine, Tyr—tyrosine, L-DOPA—L-3,4-Dihydroxyphenylalanine, DA—dopamine, NE—norepinephrine, EPI—epinephrine, DOPAL—3,4-dihydroxyphenylacetaldehyde, DOPAC—3,4-dihydroxyphenylacetic acid, DOPEGAL—3,4-Dihydroxyphenylglycolaldehyde, HVA—homovanillic acid, VMA—vanillylmandelic acid, DHPAA—3,4-Dihydroxyphenylacetic Acid. Enzymes (yellow ovals): PAH—phenylalanine hydroxylase, TH—tyrosine hydroxylase, AADC—aromatic L-amino acid decarboxylase, DDC—DOPA decarboxylase, DBH—dopamine β-hydroxylase, PNMT—phenylethanolamine N-methyltransferase, ALDH—aldehyde dehydrogenase, COMT—catechol-O-methyltransferase, MAO—monoamine oxidase. Normal enzymatic activity. Inhibited enzymatic activity.

Considering these findings, a possible hypothesis for serotonergic dysregulation in ADHD could be genetic changes due to polymorphisms in TPH2, DDC, and ASMT that hinder the conversion of tyrosine into serotonin and melatonin or tryptophan in dopamine or epigenetic changes induced by stress in prenatal or early postnatal stages in these pathways in the central nervous system, leaving more available tryptophan at the peripheral level to be metabolized through the kynurenine pathway, favoring an imbalance between neurotoxic and neuroprotective metabolites that can cross the blood–brain barrier. Additionally, these inflammatory states affect the functioning of KMO and/or KAT mediated by brain glial cells, increasing inflammation (see Figure 2a).

#### 3.1.2. Tyrosine Pathway and ADHD

Dopamine in the brain is synthesized from the enzyme tyrosine hydroxylase (TH), which converts it into levodopa (L-DOPA) with the help of tetrahydrobiopterin (BHA) cofactor and iron; L-DOPA is then decarboxylated by DDC to form dopamine, a neurotransmitter involved in coordinating movement and regulating mood, hormonal state, and even cardiovascular function [57]. It can also be indirectly produced from phenylalanine, which is catalyzed by the enzyme phenylalanine hydroxylase (PAH) to form tyrosine [58]. Through the action of dopamine beta-hydroxylase (DBH), tyrosine in the nervous and endocrine system is converted into norepinephrine and then into adrenaline by phenylethanolamine-N-methyltransferase (PNMT), the latter of which is an important hormone in the HPA system and fight-or-flight response [59]. Once it has exerted its function in the CNS, it is degraded by monoamine oxidase (MAO), aldehyde dehydrogenase (ALDH), and catechol-O-methyltransferase (COMT), producing 3,4-dihydroxyphenylacetaldehyde (DOPAL), 3,4-dihydroxyphenylacetic acid (DOPAC), and homovanillic acid (HVA), respectively [60].

Recessive mutations in genes encoding enzymes in the dopamine pathway have been described [61]. Specific polymorphisms in the genes contributing to TH expression have not been found, but animal model reports have shown adequate tyrosine levels but low dopamine levels in rats with motor hyperactivity, whose behavior improves after L-DOPA administration [62]. Genetic research has shown altered MAO and COMT enzyme function but not ALDH in ADHD patients. In fact, in vitro studies suggested that methylphenidate increases the catalytic activity of TH and MAO [63].

Another important marker of CNS function in ADHD patients is vanillylmandelic acid. The mediation exerted by COMT and MAO enzymes in both the norepinephrine and adrenaline pathways to produce this final metabolite allows for a measure of the intermediate dopamine catalysis system function and can be used as a marker of proper enzyme regulation and an indirect measure of catecholamine levels in the CNS [60,64].

Inflammatory states have been associated with alterations in tyrosine metabolism, which can affect neurotransmission. This may occur due to disruptions in the function of tyrosine hydroxylase [58] or reduced availability of tyrosine as a result of oxidative stress [65]. Although elevated inflammatory markers and deficiencies in tyrosine metabolism have been found in individuals with ADHD, further research is needed to clarify the precise nature of these relationships (see Figure 2b).

This background suggests that aromatic amino acids, such as phenylalanine, tyrosine, and tryptophan, are essential for the production of neurotransmitters that regulate attention and behavior. Brain inflammation can disrupt the metabolism of these amino acids, affecting the production of key neurotransmitters and contributing to ADHD symptoms, such as attention difficulties and impulsivity. Investigating this relationship is crucial for better understanding the impact of inflammation on ADHD and for developing more targeted treatments. Metabolomics, by providing a detailed analysis of amino acid metabolism and its alterations, is a key tool for unraveling these mechanisms and advancing personalized therapy.

### 3.2. Fatty Acids and ADHD

Regarding fatty acids, the main results showed a downregulation of omega-3 and an imbalance between polyunsaturated fatty acids 3 and 6. However, the current research focused on the pediatric population, and we found only two studies in adults. One of them reported that compared to controls, patients over 18 years old with ADHD had lower levels of polyunsaturated acids and higher levels of monounsaturated acids; they also reported lower DHA levels in erythrocyte membranes [66]. This finding also reported lower levels of omega-3 in red blood cell membranes in this same population type [33]. This omega-3 decrease does not seem to respond solely to dietary deficiency, as the researchers analyzed food intake and nutrient levels in children, finding that although there were no differences in alimentary patterns between cases and controls, there were lower levels of polyunsaturated fatty acids [33].

Moreover, research conducted in countries with high fish consumption, such as Japan, revealed that patients with ADHD had lower levels of omega-3 fatty acids and an imbalanced omega-3/omega-6 ratio [38]. It has been theorized that excessive omega-3 fatty acid metabolism could occur in patients with ADHD due to inflammatory processes, nervous system functions, and stress responses [7,67]. Studies have even reported that diets rich in inflammatory fatty acids alter the function of the dopaminergic system in the brain, which is also related to alterations in genes associated with the dopaminergic system, such as COMT [68].

Furthermore, the levels of these metabolites could be associated with ADHD symptoms; positive correlations have been found between polyunsaturated fatty acids and executive functions, while monounsaturated fats are negatively associated with cognitive measures [69]. No associations were found with saturated fatty acids [36]. Additionally, recent systematic reviews have shown that a better treatment response is observed in people with EPA deficiency; however, a recent meta-analysis considered the evidence of cognitive change after supplementation to be marginal [70], even in animal models [71].

These findings align with results published in preclinical studies, where diets deficient in DHA have been indicated to decrease dopamine concentrations in the prefrontal cortex. Conversely, diets rich in EPA and DHA increase dopamine concentrations in this region [72] by promoting inadequate storage of newly synthesized dopamine and reducing the pool of dopaminergic vesicles [73]. This has also been reported in molecular simulation research, which revealed a direct association between brain levels of polyunsaturated fatty acids and dopamine transmission, apparently by enriching the membrane with DHA, which enhances ligand binding to the D2 dopamine receptor [74].

Despite the clear evidence linking omega-3 deficiency, particularly omega-3/omega-6 imbalance, with ADHD and the potential use of this measure as a low-cost biomarker, no studies have yet identified the expected differences concerning sex and age. These investigations are crucial for obtaining better metabolite sensitivity as a biomarker, as they could reveal how these variables affect omega-3 expression or metabolism and, consequently, its relationship with brain function.

Brain inflammation can alter amino acid metabolism, affecting neurotransmitter synthesis and contributing to ADHD symptoms. Fatty acids play a crucial role in this dynamic; low levels of omega-3 are associated with reduced membrane fluidity, altered neurotransmitter reception, weakened synapses, and changes in amino acid bioavailability. The current review reveals that omega-3 levels are significantly lower in ADHD cases compared to controls, contributing to increased inflammation and neurological dysregulation.

## 4. Conclusions and Future Directions

This review makes a valuable contribution to understanding the potential alterations in metabolic pathways associated with ADHD. In addition to advancing knowledge of the disorder’s pathophysiology mechanisms, its findings could help differentiate distinct phenotypes of the syndrome, thereby improving diagnostic, monitoring and therapeutic strategies.

The importance of measuring precursors, intermediates, end products, and degradation by-products of dopamine and serotonin is highlighted across the scoping review. Alterations in this pathway, due to enzymatic dysregulation in TPH2 [35,42], AANAT [49], and ASMT [50], may lead to increased production of QUIN and other neurotoxic compounds, which can trigger inflammatory states [75,76,77]; these states may further impair the activity of glial cell-regulated enzymes KMO and KAT, intensifying inflammation [38]. The inflammatory processes interfere with amino acid and fatty acid metabolism, impairing neurotransmitter synthesis and potentially exacerbating ADHD symptoms [21]. Variability in these biochemical pathways, especially in the context of inflammation, may account for the variability observed in ADHD phenotypes. It has been hypothesized that persistent inflammatory processes and/or stress responses could contribute to excessive omega-3 consumption, which, in turn, might increase inflammation [7] and disrupt dopaminergic system function [78]. In particular, omega-3 plays a crucial role in this process; low omega-3 levels have been associated with reduced membrane fluidity, altered neurotransmitter activity, weakened synaptic connections, and limited availability of essential amino acids [7,79]. This review emphasizes that individuals with ADHD, both children and adults, exhibit significantly lower omega-3 levels compared to control groups, along with an imbalance between polyunsaturated fatty acids omega-3 and omega-6.

Nevertheless, the available evidence in ADHD metabolomics is insufficient, highlighting the need for a comprehensive metabolite profile to trace pathophysiological pathways and assess their involvement in ADHD. While metabolomics holds great promise, it remains an emerging field. Conducting large-scale metabolite panel analyses using rigorous statistical methods could strengthen diagnostic frameworks for this highly heterogeneous disorder. Furthermore, acknowledging the significant role of genetic and epigenetic factors in these biochemical disturbances underscores the importance of integrating metabolomics with other omics-based technologies. This integrative approach offers the potential for a more thorough understanding of ADHD, paving the way for more accurate diagnostic criteria and personalized therapeutic strategies.

Future research should focus on well-designed experimental studies with large and diverse samples, employing untargeted approaches across various biofluids. Providing detailed cognitive and neuropsychological data will aid in identifying associations between clinical phenotypes and metabolic profiles. Additionally, these should evaluate the biochemical pathways and reactions across entire metabolic pathways, including not only the initial metabolites but also their intermediates and downstream products, to gain a comprehensive understanding of the underlying mechanisms. To further enhance this approach, integrating “omics” disciplines could provide a more comprehensive framework for analyzing metabolic networks and their interactions, offering deeper insights into the complexity of these biological systems.

Some limitations of this review must be noted. First, most of the included studies reported a higher prevalence of male participants, consistent with known epidemiological patterns of ADHD. Second, the small number of studies and their methodological differences—including variations in participant age, sex, control group selection, and study design—pose challenges to making consistent comparisons. Finally, as this is still an emerging field, many studies using high-sensitivity detection techniques remain observational. This review excluded recent works that analyzed metabolite panels but lacked appropriate control groups, highlighting the need for higher methodological standards in future research to produce more reliable and generalizable conclusions. Future reviews should also incorporate in silico modeling, which could enable more robust conclusions by simulating metabolic pathways and predicting potential outcomes based on existing data.

## Supplementary Materials

The following supporting information can be downloaded at: https://www.mdpi.com/article/10.3390/metabo15020133/s1, Table S1: Search criteria.

## References

[1] Drechsler R., Brem S., Brandeis D., Grünblatt E., Berger G., Walitza S. (2020). ADHD: Current concepts and treatments in children and adolescents. Neuropediatrics.

[2] Polanczyk G.V., Willcutt E.G., Salum G.A., Kieling C., Rohde L.A. (2014). ADHD prevalence estimates across three decades: An updated systematic review and meta-regression analysis. Int. J. Epidemiol..

[3] Thomas R., Sanders S., Doust J., Beller E., Glasziou P. (2015). Prevalence of attention-deficit/hyperactivity disorder: A systematic review and meta-analysis. Pediatrics.

[4] Green A., Baroud E., DiSalvo M., Faraone S.V., Biederman J. (2022). Examining the impact of ADHD polygenic risk scores on ADHD and associated outcomes: A systematic review and meta-analysis. J. Psychiatr. Res..

[5] Biederman J., Petty C.R., Woodworth K.Y., Lomedico A., Hyder L.L., Faraone S.V. (2012). Adult outcome of attention-deficit/hyperactivity disorder: A controlled 16-year follow-up study. J. Clin. Psychiatry.

[6] Elhady M., Elattar R.S., Elaidy A.M.A., Abdallah N.A., Elmalt H.A. (2022). Role of inflammation in childhood epilepsy and ADHD comorbidity. Appl. Neuropsychol. Child.

[7] Donev R., Thome J. (2010). Inflammation: Good or bad for ADHD?. ADHD Atten. Deficit Hyperact. Disord..

[8] Anand N.S., Ji Y., Wang G., Hong X., van der Rijn M., Riley A., Pearson C., Zuckerman B., Wang X. (2021). Maternal and cord plasma branched-chain amino acids and child risk of attention-deficit hyperactivity disorder: A prospective birth cohort study. J. Child. Psychol. Psychiatry.

[9] Skalny A.V., Mazaletskaya A.L., Zaitseva I.P., Skalny A.A., Spandidos D.A., Tsatsakis A., Lobanova Y.N., Skalnaya M.G., Aschner M., Tinkov A.A. (2021). Alterations in serum amino acid profiles in children with attention deficit/hyperactivity disorder. Biomed. Rep..

[10] Robberecht H., Verlaet A.A.J., Breynaert A., de Bruyne T., Hermans N. (2020). Magnesium, Iron, Zinc, copper and selenium status in attention-deficit/hyperactivity disorder (ADHD). Molecules.

[11] Rucklidge J.J. (2010). Gender Differences in Attention-Deficit/Hyperactivity Disorder. Psychiatric. Clinic..

[12] Sinn N., Bryan J. (2007). Effect of Supplementation with Polyunsaturated Fatty Acids and Micronutrients on Learning and Behavior Problems Associated with Child ADHD. J. Dev. Behav. Pediatr..

[13] Padilla M.F.Q., Lázaro J.C.F., Rodríguez N.G., Sánchez H.N. (2021). Propuesta de un modelo predictivo para el déficit de atención en adultos jóvenes de Colombia y sus posibles comorbilidades. Alcances en Neurociencias Cognitivas. Tomo 1: Modelo Para la Fundamentación de la Línea de Investigación en Neurociencias y Neurodesarrollo.

[14] Sethi S., Brietzke E. (2015). Omics-based biomarkers: Application of metabolomics in neuropsychiatric disorders. Int. J. Neuropsychopharmacol..

[15] Bonvicini C., Faraone S.V., Scassellati C. (2016). Attention-deficit hyperactivity disorder in adults A systematic review and meta-analysis of genetic, pharmacogenetic and biochemical studies. Mol. Psychiatry.

[16] Bose S., Mandal S., Khan R., Maji H.S., Ashique S. (2023). Current Landscape on Development of Phenylalanine and Toxicity of its Metabolites—A Review. Curr. Drug Saf..

[17] Banerjee E., Nandagopal K. (2015). Does serotonin deficit mediate susceptibility to ADHD?. Neurochem. Int..

[18] Hou Y., Xiong P., Gu X., Huang X., Wang M., Wu J. (2018). Association of Serotonin Receptors with Attention Deficit Hyperactivity Disorder: A Systematic Review and Meta-analysis. Curr. Med. Sci..

[19] Leshem R., Bar-Oz B., Diav-Citrin O., Gbaly S., Soliman J., Renoux C., Matok I. (2021). Selective Serotonin Reuptake Inhibitors (SSRIs) and Serotonin Norepinephrine Reuptake Inhibitors (SNRIs) During Pregnancy and the Risk for Autism spectrum disorder (ASD) and attention deficit hyperactivity disorder (ADHD) in the Offspring: A True Effect or a Bias? A Systematic Review & Meta-Analysis. Curr. Neuropharmacol..

[20] Cannon Homaei S., Barone H., Kleppe R., Betari N., Reif A., Haavik J. (2022). ADHD symptoms in neurometabolic diseases: Underlying mechanisms and clinical implications. Neurosci. Biobehav. Rev..

[21] Chang J.P.C., Su K.P., Mondelli V., Pariante C.M. (2018). Omega-3 Polyunsaturated Fatty Acids in Youths with Attention Deficit Hyperactivity Disorder: A Systematic Review and Meta-Analysis of Clinical Trials and Biological Studies. Neuropsychopharmacology.

[22] Widenhorn-Müller K., Schwanda S., Scholz E., Spitzer M., Bode H. (2014). Effect of supplementation with long-chain ω-3 polyunsaturated fatty acids on behavior and cognition in children with attention deficit/hyperactivity disorder (ADHD): A randomized placebo-controlled intervention trial. Prostaglandins Leukot. Essent. Fatty Acids.

[23] Johnson M., Månsson J.E., Östlund S., Fransson G., Areskoug B., Hjalmarsson K., Landgren M., Kadesjö B., Gillberg C. (2012). Fatty acids in ADHD: Plasma profiles in a placebo-controlled study of Omega 3/6 fatty acids in children and adolescents. ADHD Atten. Deficit Hyperact. Disord..

[24] Hariri M., Jazayery A., Jalali M., Rahimi A., Abdohahian E. (2012). Effect of omega-3 supplementation on hyperactivity, oxidative stress in children with attention-deficit-hyperactivity disorder. Malays. J. Nutr..

[25] Bloch M.H., Qawasmi A. (2011). Omega-3 fatty acid supplementation for the treatment of children with attention-deficit/hyperactivity disorder symptomatology: Systematic review and meta-analysis. J. Am. Acad. Child Adolesc. Psychiatry.

[26] Tian X., Liu X., Wang Y., Liu Y., Ma J., Sun H., Li J., Tang X., Guo Z., Sun W. (2022). Urinary Metabolomic Study in a Healthy Children Population and Metabolic Biomarker Discovery of Attention-Deficit/Hyperactivity Disorder (ADHD). Front. Psychiatry.

[27] Sinningen K., Emons B., Böhme P., Juckel G., Hanusch B., Beckmann B., Tsikas D., Lücke T. (2023). L-Arginine/nitric oxide pathway and oxidative stress in adults with ADHD: Effects of methylphenidate treatment. Nitric Oxide.

[28] Raghavan R., Anand N.S., Wang G., Hong X., Pearson C., Zuckerman B., Xie H., Wang X. (2022). Association between cord blood metabolites in tryptophan pathway and childhood risk of autism spectrum disorder and attention-deficit hyperactivity disorder. Transl. Psychiatry.

[29] Gyamfi J., Kim J., Choi J. (2022). Cancer as a Metabolic Disorder. Int. J. Mol. Sci..

[30] Bergwerff C.E., Luman M., Blom H.J., Oosterlaan J. (2016). No tryptophan, tyrosine and phenylalanine abnormalities in children with attention-deficit/hyperactivity disorder. PLoS ONE.

[31] Ildiz G.O., Karadag A., Kaygisiz E., Fausto R. (2021). PLS-DA model for the evaluation of attention deficit and hyperactivity disorder in children and adolescents through blood serum FTIR spectra. Molecules.

[32] Wang L.J., Chou W.J., Tsai C.S., Lee M.J., Lee S.Y., Hsu C.W., Hsueh P.C., Wu C.C. (2021). Novel plasma metabolite markers of attention-deficit/hyperactivity disorder identified using high-performance chemical isotope labelling-based liquid chromatography-mass spectrometry. World J. Biol. Psychiatry.

[33] Chen J.R., Hsu S.F., Hsu C.D., Hwang L.H., Yang S.C. (2004). Dietary patterns and blood fatty acid composition in children with attention-deficit hyperactivity disorder in Taiwan. J. Nutr. Biochem..

[34] Young G.S., Maharaj N.J., Conquer J.A. (2004). Blood Phospholipid Fatty Acid Analysis of Adults with and Without Attention Deficit/Hyperactivity Disorder. Lipids.

[35] Joshi K., Lad S., Kale M., Patwardhan B., Mahadik S.P., Patni B., Chaudhary A., Bhave S., Pandit A. (2006). Supplementation with flax oil and vitamin C improves the outcome of Attention Deficit Hyperactivity Disorder (ADHD). Prostaglandins Leukot. Essent. Fatty Acids.

[36] Laasonen M., Hokkanen L., Leppämäki S., Tani P., Erkkilä A.T. (2009). Project DyAdd: Fatty acids and cognition in adults with dyslexia, ADHD, or both. Prostaglandins Leukot. Essent. Fatty Acids.

[37] Gustafsson P.A., Birberg-Thornberg U., Duchén K., Landgren M., Malmberg K., Pelling H., Strandvik B., Karlsson T. (2010). EPA supplementation improves teacher-rated behaviour and oppositional symptoms in children with ADHD. Acta Paediatrica..

[38] Yonezawa K., Nonaka S., Iwakura Y., Kusano Y., Funamoto Y., Kanchi N., Yamaguchi N., Kusumoto Y., Imamura A., Ozawa H. (2018). Investigation into the plasma concentration of ω3 polyunsaturated fatty acids in Japanese attention-deficit hyperactivity disorder patients. J. Neural Transm..

[39] Page M.J., McKenzie J.E., Bossuyt P.M., Boutron I., Hoffmann T.C., Mulrow C.D., Shamseer L., Tetzlaff J.M., Akl E.A., Brennan S.E. (2021). The PRISMA 2020 statement: An updated guideline for reporting systematic reviews. BMJ.

[40] Swami T., Weber H.C. (2018). Updates on the biology of serotonin and tryptophan hydroxylase. Curr. Opin. Endocrinol. Diabetes Obes..

[41] Sheehan K., Lowe N., Kirley A., Mullins C., Fitzgerald M., Gill M., Hawi Z. (2005). Tryptophan hydroxylase 2 (TPH2) gene variants associated with ADHD. Mol. Psychiatry.

[42] Akhrif A., Romanos M., Peters K., Furtmann A.K., Caspers J., Lesch K.P., Meisenzahl-Lechner E.M., Neufang S. (2023). Serotonergic modulation of normal and abnormal brain dynamics: The genetic influence of the TPH2 G-703T genotype and DNA methylation on wavelet variance in children and adolescents with and without ADHD. PLoS ONE.

[43] Akhrif A., Roy A., Peters K., Lesch K.P., Romanos M., Schmitt-Böhrer A., Neufang S. (2021). REVERSE phenotyping—Can the phenotype following constitutive Tph2 gene inactivation in mice be transferred to children and adolescents with and without adhd?. Brain Behav..

[44] Baehne C.G., Ehlis A.C., Plichta M.M., Conzelmann A., Pauli P., Jacob C., Gutknecht L., Lesch K.P., Fallgatter A.J. (2009). Tph2 gene variants modulate response control processes in adult ADHD patients and healthy individuals. Mol. Psychiatry.

[45] Pulver A., Kiive E., Harro J. (2020). Reward sensitivity, affective neuroscience personality, symptoms of attention-deficit/hyperactivity disorder, and TPH2-703G/T (rs4570625) genotype. Acta Neuropsychiatr..

[46] Roberts K.M., Fitzpatrick P.F. (2013). Mechanisms of tryptophan and tyrosine hydroxylase. IUBMB Life.

[47] Blau N., Pearson T.S., Kurian M.A. (2023). Aromatic L-Amino Acid Decarboxylase Deficiency.

[48] Moskaleva P.V., Shnayder N.A., Nasyrova R.F. (2021). Association of polymorphic variants of ddc (Aadc), aanat and asmt genes encoding enzymes for melatonin synthesis with the higher risk of neuropsychiatric disorders. Zhurnal Nevrol. Psihiatr. Im. S.S. Korsakova.

[49] Holvoet E., Gabriëls L. (2013). Verstoorde slaap bij kinderen met ADHD: Heeft melatonine een plaats in de behandeling?. Tijdschr. Voor Psychiatr..

[50] Chaste P., Clement N., Botros H.G., Guillaume J.L., Konyukh M., Pagan C., Scheid I., Nygren G., Anckarsäter H., Rastam M. (2011). Genetic variations of the melatonin pathway in patients with attention-deficit and hyperactivity disorders. J. Pineal Res..

[51] Trent S., Fry J.P., Ojarikre O.A., Davies W. (2014). Altered brain gene expression but not steroid biochemistry in a genetic mouse model of neurodevelopmental disorder. Mol. Autism.

[52] Badawy A.A.B. (2020). Kynurenine pathway and human systems. Exp. Gerontol..

[53] Park J.H. (2022). Potential Inflammatory Biomarker in Patients with Attention Deficit Hyperactivity Disorder. Int. J. Mol. Sci..

[54] Kamradt J.M., Momany A.M., Nikolas M.A. (2018). A meta-analytic review of the association between cortisol reactivity in response to a stressor and attention-deficit hyperactivity disorder. ADHD Atten. Deficit Hyperact. Disord..

[55] Myint A.M., Kim Y.K. (2014). Network beyond IDO in psychiatric disorders: Revisiting neurodegeneration hypothesis. Prog. Neuro-Psychopharmacol. Biol. Psychiatry.

[56] Evangelisti M., De Rossi P., Rabasco J., Donfrancesco R., Lionetto L., Capi M., Sani G., Simmaco M., Nicoletti F., Villa M.P. (2017). Changes in serum levels of kynurenine metabolites in paediatric patients affected by ADHD. Eur. Child Adolesc. Psychiatry.

[57] Klein M.O., Battagello D.S., Cardoso A.R., Hauser D.N., Bittencourt J.C., Correa R.G. (2019). Dopamine: Functions, Signaling, and Association with Neurological Diseases. Cell. Mol. Neurobiol..

[58] Stevenson M., McNaughton N. (2013). A comparison of phenylketonuria with attention deficit hyperactivity disorder: Do markedly different aetiologies deliver common phenotypes?. Brain Res. Bull..

[59] Van West D., Claes S., Deboutte D. (2009). Differences in hypothalamic-pituitary-adrenal axis functioning among children with ADHD predominantly inattentive and combined types. Eur. Child Adolesc. Psychiatry.

[60] Liu L., Cheng J., Su Y., Ji N., Gao Q., Li H., Yang L., Sun L., Qian Q., Wang Y. (2018). Deficiency of Sustained Attention in ADHD and Its Potential Genetic Contributor MAOA. J. Atten. Disord..

[61] Cabana-Domínguez J., Torrico B., Reif A., Fernàndez-Castillo N., Cormand B. (2022). Comprehensive exploration of the genetic contribution of the dopaminergic and serotonergic pathways to psychiatric disorders. Transl. Psychiatry.

[62] Yamaguchi T., Nagasawa M., Ikeda H., Kodaira M., Minaminaka K., Chowdhury V.S., Yasuo S., Furuse M. (2017). Manipulation of dopamine metabolism contributes to attenuating innate high locomotor activity in ICR mice. Behav. Brain Res..

[63] Bartl J., Palazzesi F., Parrinello M., Hommers L., Riederer P., Walitza S., Grünblatt E. (2017). The impact of methylphenidate and its enantiomers on dopamine synthesis and metabolism in vitro. Prog. Neuro-Psychopharmacol. Biol. Psychiatry.

[64] Neri L., Marziani B., Sebastiani P., Del Beato T., Colanardi A., Legge M.P., Aureli A. (2024). Aggressiveness in Italian Children with ADHD: MAOA Gene Polymorphism Involvement. Diseases.

[65] Fitzpatrick P.F. (2023). The aromatic amino acid hydroxylases: Structures, catalysis, and regulation of phenylalanine hydroxylase, tyrosine hydroxylase, and tryptophan hydroxylase. Arch. Biochem. Biophys..

[66] Young G.S., Conquer J.A., Thomas R. (2005). Effect of randomized supplementation with high dose olive, flax or fish oil on serum phospholipid fatty acid levels in adults with attention deficit hyperactivity disorder. Reprod. Nutr. Dev..

[67] Saccaro L.F., Schilliger Z., Perroud N., Piguet C. (2021). Inflammation, anxiety, and stress in attention-deficit/ hyperactivity disorder. Biomedicines.

[68] Sun H.X., Wang D.R., Ye C.B., Hu Z.Z., Wang C.Y., Huang Z.L., Yang S.R. (2017). Activation of the ventral tegmental area increased wakefulness in mice. Sleep Biol. Rhythm..

[69] Döpfner M., Dose C., Breuer D., Heintz S., Schiffhauer S., Banaschewski T. (2021). Efficacy of Omega-3/Omega-6 Fatty Acids in Preschool Children at Risk of ADHD: A Randomized Placebo-Controlled Trial. J. Atten. Disord..

[70] Händel M.N., Rohde J.F., Rimestad M.L., Bandak E., Birkefoss K., Tendal B., Lemcke S., Callesen H.E. (2021). Efficacy and safety of polyunsaturated fatty acids supplementation in the treatment of attention deficit hyperactivity disorder (Adhd) in children and adolescents: A systematic review and meta-analysis of clinical trials. Nutrients.

[71] Transler C., Mitchell S., Eilander A. (2013). Could Polyunsaturated Fatty Acids Deficiency Explain Some Dysfunctions Found in ADHD? Hypotheses From Animal Research. J. Atten. Disord..

[72] Zimmer-Gembeck M.J., McKay A., Webb H.J. (2019). The Food-Related Parenting Context: Associations with Parent Mindfulness and Children’s Temperament. Mindfulness.

[73] Dervola K.S., Roberg B.T., Wøien G., Bogen I.L., Sandvik T.H., Sagvolden T., Drevon C.A., Johansen E.B., Walaas S.I. (2012). Marine omega-3 polyunsaturated fatty acids induce sex-specific changes in reinforcer-controlled behaviour and neurotransmitter metabolism in a spontaneously hypertensive rat model of ADHD. Behav. Brain Funct..

[74] Jobin M.L., De Smedt-Peyrusse V., Ducrocq F., Baccouch R., Oummadi A., Pedersen M.H., Medel-Lacruz B., Angelo M.F., Villette S., Van Delft P. (2023). Impact of membrane lipid polyunsaturation on dopamine D2 receptor ligand binding and signaling. Mol. Psychiatry.

[75] Topal Z., Tufan A.E., Karadag M., Gokcen C., Akkaya C., Sarp A.S., Bahsi I., Kilinc M. (2022). Evaluation of peripheral inflammatory markers, serum B12, folate, ferritin levels and clinical correlations in children with autism spectrum disorder (ASD) and attention deficit hyperactivity disorder (ADHD). Nord. J. Psychiatry.

[76] Allred E.N., Dammann O., Fichorova R.N., Hooper S.R., Hunter S.J., Joseph R.M., Kuban K., Leviton A., O’Shea T.M., Scott M.N. (2017). Systemic Inflammation during the First Postnatal Month and the Risk of Attention Deficit Hyperactivity Disorder Characteristics among 10 year-old Children Born Extremely Preterm. J. Neuroimmune Pharmacol..

[77] Chang J.P.C., Su K.P., Mondelli V., Pariante C.M. (2021). Cortisol and inflammatory biomarker levels in youths with attention deficit hyperactivity disorder (ADHD): Evidence from a systematic review with meta-analysis. Transl. Psychiatry.

[78] Healy-Stoffel M., Levant B. (2018). N-3 (Omega-3) Fatty Acids: Effects on Brain Dopamine Systems and Potential Role in the Etiology and Treatment of Neuropsychiatric Disorders. CNS Neurol. Disord. Drug Targets.

[79] Predescu E., Vaidean T., Rapciuc A.M., Sipos R. (2021). Metabolomic Markers in Attention-Deficit/Hyperactivity Disorder (ADHD) among Children and Adolescents—A Systematic Review. Int. J. Mol. Sci..

