# Supplementary material for: Metabolomic Insights into Attention Deficit Hyperactivity Disorder: A Scoping Review
Source: Metabolites. 2025 Feb 16;15(2):133. doi: 10.3390/metabo15020133 (PMC11857725; doi:10.3390/metabo15020133)
Supplement: Supplementary file 1 [file metabolites-15-00133-s001.zip › metabolites-3355028-supplementary.pdf]

Supplementary Materials:

Table S1: Search criteria

Database	Search Criteria	Number
PubMed	((((("metabolome"[MeSH Terms]) OR ("biomarkers"[MeSH Terms])) OR ("lipidomics"[MeSH Terms])) AND ("attention deficit and disruptive behavior disorders/metabolism"[MeSH Terms])) OR ("attention deficit and disruptive behavior disorders/blood"[MeSH Terms])) OR ("attention deficit and disruptive behavior disorders/blood"[MeSH Terms] OR "attention deficit and disruptive behavior disorders/analysis"[MeSH Terms])	431
Web of Science	(((((((TI=(metabolomics)) OR TI=(metabolome)) OR TI=(biomarkers)) OR TI=(lipidomics)) AND TI=(Attention Deficit Disorders with Hyperactivity)) OR TI=(Hyperkinetic Syndrome))) NOT ALL=(ANIMALS)	39
Scopus	TITLE-ABS-KEY (metabolome) OR TITLE-ABS-KEY (metabolomics) OR TITLE-ABS-KEY (lipidomics) TITLE-ABS-KEY (biomarkers) AND TITLE-ABS-KEY (*attention AND deficit AND disorders AND with AND hyperactivity*) OR TITLE-ABS-KEY (hyperkinetic AND syndrome OR attention AND deficit) AND NOT (animal	24
